# Standards of fracture care in polytrauma: results of a Europe-wide survey by the ESTES polytrauma section

**DOI:** 10.1007/s00068-022-02126-3

**Published:** 2022-10-13

**Authors:** Julian Scherer, Raul Coimbra, Diego Mariani, Luke Leenen, Radko Komadina, Ruben Peralta, Luka Fattori, Ingo Marzi, Klaus Wendt, Christine Gaarder, Hans-Christoph Pape, Roman Pfeifer

**Affiliations:** 1 Department of Trauma Surgery, University Hospital Zurich, University of Zurich, Raemistr. 100, 8091 Zurich, Switzerland; 2 Riverside University Health System and Loma Linda University, Riverside, CA USA; 3 Department of Emergency General Surgery, Legnano Hospital, ASST Ovest Milanese, Legnano, MI Italy; 4 Department of Surgery, University Medical Center Utrecht, Utrecht, The Netherlands; 5 Department of Traumatology, General and Teaching Hospital Celje, Medical Faculty Ljubljana University, 3000 Celje, Slovenia; 6 Surgical Department (Hamad General Hospital), Hamad Medical Corporation, HMC, Doha, Qatar; 7 Department of Surgery, San Gerardo Hospital, University of Milan Bicocca, G.B. Pergolesi 33, Monza, Italy; 8 Department of Trauma, Hand and Reconstructive Surgery, University Hospital Frankfurt, Goethe-University, Frankfurt, Germany; 9 Department of Trauma Surgery, University Medical Center Groningen (UMCG), Hanzeplein 1, 9700 RB Groningen, The Netherlands; 10 Department of Traumatology, Oslo University Hospital Ullevål, Kirkeveien 166, 0450 Oslo, Norway

**Keywords:** Major fractures, Polytrauma, Damage control orthopaedics, Fracture care

## Abstract

**Introduction:**

Fixation of major fractures plays a pivotal role in the surgical treatment of polytrauma patients. In addition to ongoing discussions regarding the optimal timing in level I trauma centers, it appears that the respective trauma systems impact the implementation of both, damage control and safe definitive surgery strategies. This study aimed to assess current standards of polytrauma treatment in a Europe-wide survey.

**Methods:**

A survey, developed by members of the polytrauma section of ESTES, was sent online via SurveyMonkey^®^, between July and November 2020, to 450 members of ESTES (European Society of Trauma and Emergency Surgery). Participation was voluntary and anonymity was granted. The questionnaire consisted of demographic data and included questions about the definition of “polytrauma” and the local standards for the timing of fracture fixation.

**Results:**

In total, questionnaires of 87 participants (19.3% response rate) were included. The majority of participants were senior consultants (50.57%). The mean work experience was 19 years, and on average, 17 multiple-injured patients were treated monthly. Most of the participants stated that a polytrauma patient is defined by ISS ≥ 16 (44.16%), followed by the “Berlin Definition” (25.97%). Systolic blood pressure < 90 mmHg, tachycardia or vasopressor administration (86.84%), pH deviation, base excess shift (48.68%), and lactate > 4 mmol (40.79%) or coagulopathy defined by ROTEM (40.79%) were the three most often stated indicators for shock. Local guidelines (33.77%) and the S-3 Guideline by the DGU® (23.38%) were mostly stated as a reference for the treatment of polytrauma patients. Normal coagulation (79.69%), missing administration of vasopressors (62.50%), and missing clinical signs of “SIRS” (67.19%) were stated as criteria for safe definite secondary surgery.

**Conclusion:**

Different definitions of polytrauma are used in the clinical setting. Indication for and the extent of secondary (definitive) surgery are mainly dependent on the polytrauma patient`s physiology. The «Window of Opportunity» plays a less important role in decision making.

**Supplementary Information:**

The online version contains supplementary material available at 10.1007/s00068-022-02126-3.

## Introduction

Damage control surgery is a strategy used in the treatment of severely injured patients accepted worldwide [1]. First described in the management of abdominal trauma, this concept was incorporated into the treatment of musculoskeletal trauma as “Damage Control Orthopedics” (DCO) [2–4]. Similarly, other disciplines use this approach regularly to restore patients’ physiology before definitive reconstruction. Nowadays, numerous “Damage Control” recommendations for resuscitation (DC Resuscitation), neurosurgery, ophthalmology, thoracic surgery, burns, and ICU management are available [5, 6]. It is indicated in patients that do not respond well to resuscitation, are unstable, or have specific risk factors [5]. In patients that respond adequately, safe definitive surgery of the major fracture has been recommended [7].

The incidence of temporary fracture fixation has increased, and more staged procedures are preferred than “fixation in one go.” [3] According to current strategies, multiple factors trigger a temporizing approach, i.e., the patients` physiology (shock, coagulopathy, and soft tissue injuries of the chest and extremities), and general injury severity and patterns [8]. Previous studies indicate that, in certain situations, temporary fixation is also used in physiologically stable patients [9]. In a systematic literature review and expert opinion survey by the *Polytrauma section* of ESTES, a detailed description of indications and standardized interventions for damage control orthopedics were described in polytrauma patients and in those with isolated musculoskeletal injuries [10]. The expert group suggested using the term “Musculoskeletal Temporary Surgery” (MUST Surgery) [10]. This differentiation is thought to clarify why a staged procedure was performed (local versus systemic conditions) and would facilitate the interpretation of the studies. We hypothesize that treatment strategies in polytraumatized patients have changed over the years.

Thus, the aim of this study was to understand the recent standards in fracture management in Europe, the *ESTES Polytrauma Section* developed a standardized questionnaire focusing on (1) definitions associated with a damage control strategy, (2) indications for damage control procedure, (3) indications for secondary surgeries and reconstruction, and (4) timing of definitive surgery.

## Methods

### Study design

A web-based questionnaire was developed. Pilot testing of the survey was performed by surgeons currently working in the Department of Traumatology at the University Hospital Zurich, Switzerland, and during the Polytrauma course in 2021 (www.polytraumacourse.com). The questionnaire was modified according to remarks by the participants following the pilot survey. No financial compensation was provided in exchange for participation. The questionnaire can be found as Online Appendix 1.

### Ethics approval statement

Participation was voluntary and anonymity was granted. All participants received written information explaining the aim of the study and processing of their data. No identifying data were collected. Hence, data can be assumed to be anonymous and the European data protection regulations do not apply. In addition, the local ethic committee has declared a general waiver for surveys with anonymous data. By answering the questionnaire, participants gave consent to the use of the data that they had provided.

### Survey

The complete survey consisted of 19 questions assessing the four domains described above and offered between June and November 2020. The questionnaire consisted of 4 blocks:

1. Demographics and level of education (intern, resident, attending, senior attending, head of the department). Due to the variety of definitions for the depicted levels of education among different countries, we did not define those and rather let the participants self-assess their individual level of education.
2. Questions regarding the polytrauma definition and polytrauma treatment guidelines (*n* = 4 questions)
3. Questions with focus on decision making and indications for damage control surgery in acute trauma (*n* = 6 questions).
4. Questions regarding secondary surgery and reconstruction (*n* = 4 questions).

The present survey was voluntary, and anonymity was granted. The online platform (SurveyMonkey, San Mateo, CA. USA) was used. The online survey was sent to ESTES (European Society of Trauma and Emergency Surgery) members via email (450 members in the distribution list). No reminders via email were sent. ESTES was chosen because of its European profile and representation. All data were collected anonymously in an Excel^®^ database and then statistically analyzed using SPSS® 27.

### Definitions

Berlin Definition: two injuries that are greater or equal to 3 on the AIS and one or more additional diagnoses (hypotension (systolic blood pressure ≤ 90 mm Hg,), unconsciousness (GCS score ≤ 8), acidosis (base deficit ≤ -6.0), coagulopathy (PTTQ ≥ 40 s or INR ≥ 1.4), and age (≥ 70 years) [11].

SIRS (Systemic inflammatory response syndrome) any two of the following criteria: Body temperature > 38 or < 36 °C, heart rate > 90 beats per minute, respiratory rate greater than 20 breaths/minute or partial pressure of CO_2_ less than 32 mmHg, leukocyte count greater than 12,000 or less than 4000 /microliters or over 10% immature forms or bands [12].

Window of Opportunity: Safe definite surgery after day 5–8 after polytrauma [13].

The triad of death: Shock/acidosis, coagulopathy, and hypothermia [14].

## Results:

A total of *n* = 87 (100%, 19.3% response rate) fully completed questionnaires were included. The majority of the participants were male 85.1% (*n* = 74). The mean number of years of experience in surgery was 19.3 years. Twenty (22.9%) of participants were head of the department, *n* = 44 (50.6%) were senior attendings, *n* = 16 (18.4%) attendings, and *n* = 7 (8.1%) residents.

### Definitions

**Fig. 1:**
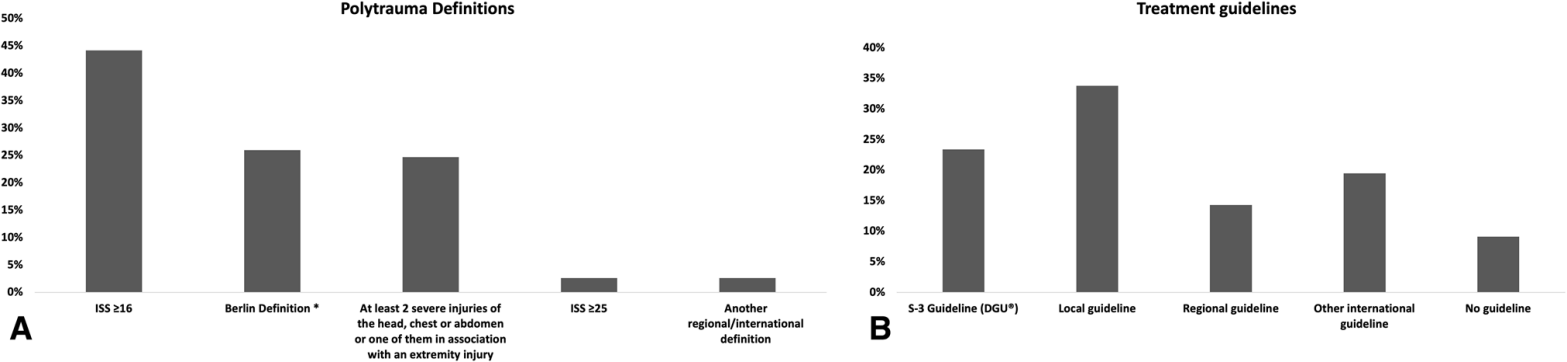
A: Polytrauma Definitions B: Treatment guidelines. *2 injuries equal or greater than AIS 3 and one or more additional diagnosis (pathological condition): systolic blood pressure ≤ 90 mmHg; GCS ≤ 8; base deficit ≤ − 6.0; PTT ≥ 40 s. /INR > 1.4; age ≥ 70 years

The distribution of used polytrauma definitions is depicted in Fig. 1A. Up to 44% (*n* = 34) of participants used the anatomic definition of ISS ≥ 16, and the “Berlin Definition” of polytrauma was mentioned by 25.9% (*n* = 20). In regard to treatment guidelines for polytrauma patients, one-third of the participants (33.77%, *n* = 26) used local hospital guidelines, and 23.38% (*n* = 18) used the S3 Guideline (DGU^®^) [15] (Fig. 1B).

### Definition of major fractures

**Fig. 2:**
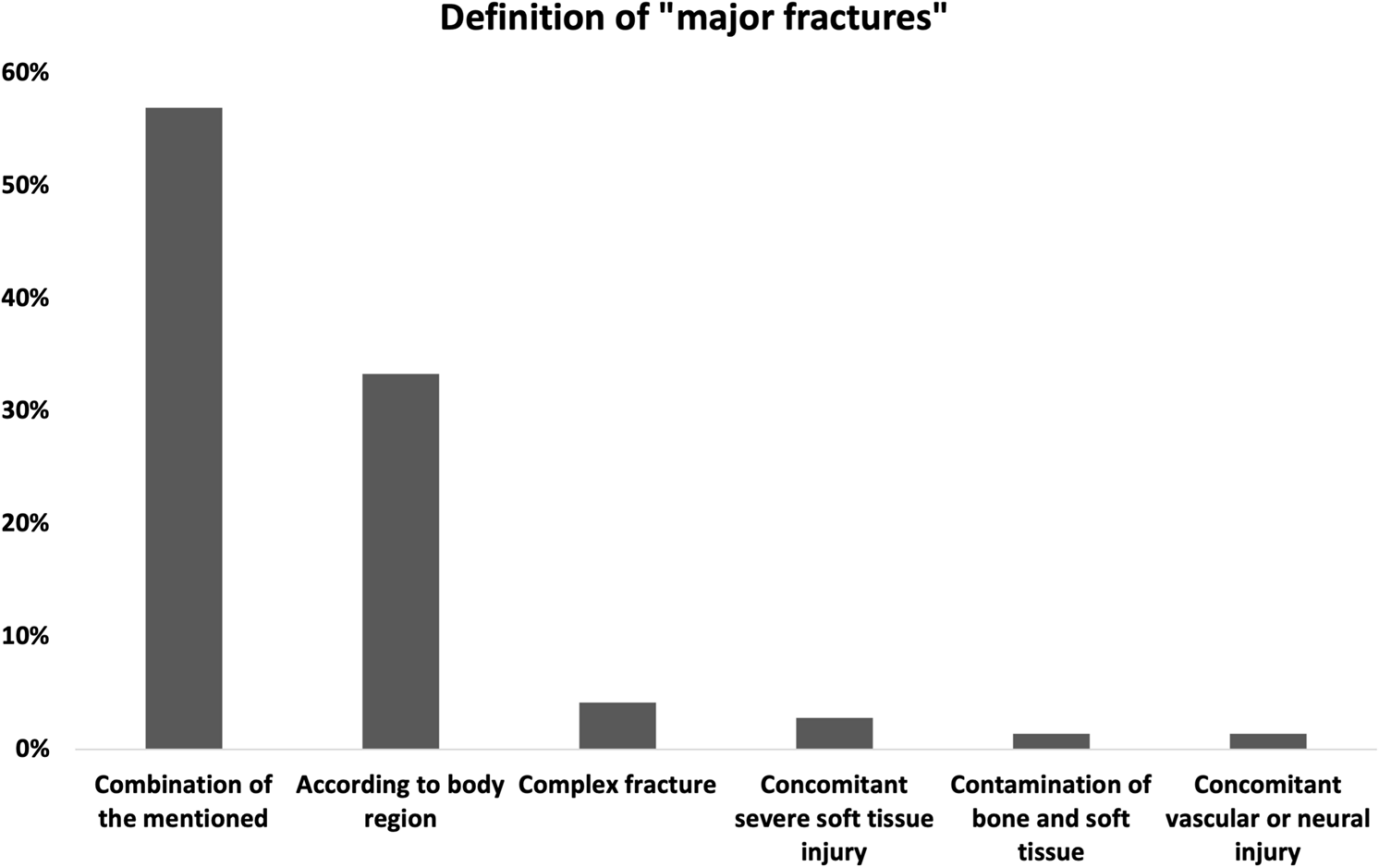
Definition of “major fractures”

Most of the surgeons (56.94%, *n* = 41) define major fractures as a combination of clinical features such as the presence of concomitant injuries (1.39%, *n* = 1), the degree of contamination of bone and soft tissue (1.39%, *n* = 1), the presence of severe soft tissue injury (2.78%, *n* = 2), and the presence of a complex fracture type (e.g., intra-articular fracture) (4.17%, *n* = 3) (Fig. 2). However, 33.33% (*n* = 24) define major fractures according to the body region.

### Indication for damage control orthopedics

**Fig. 3:**
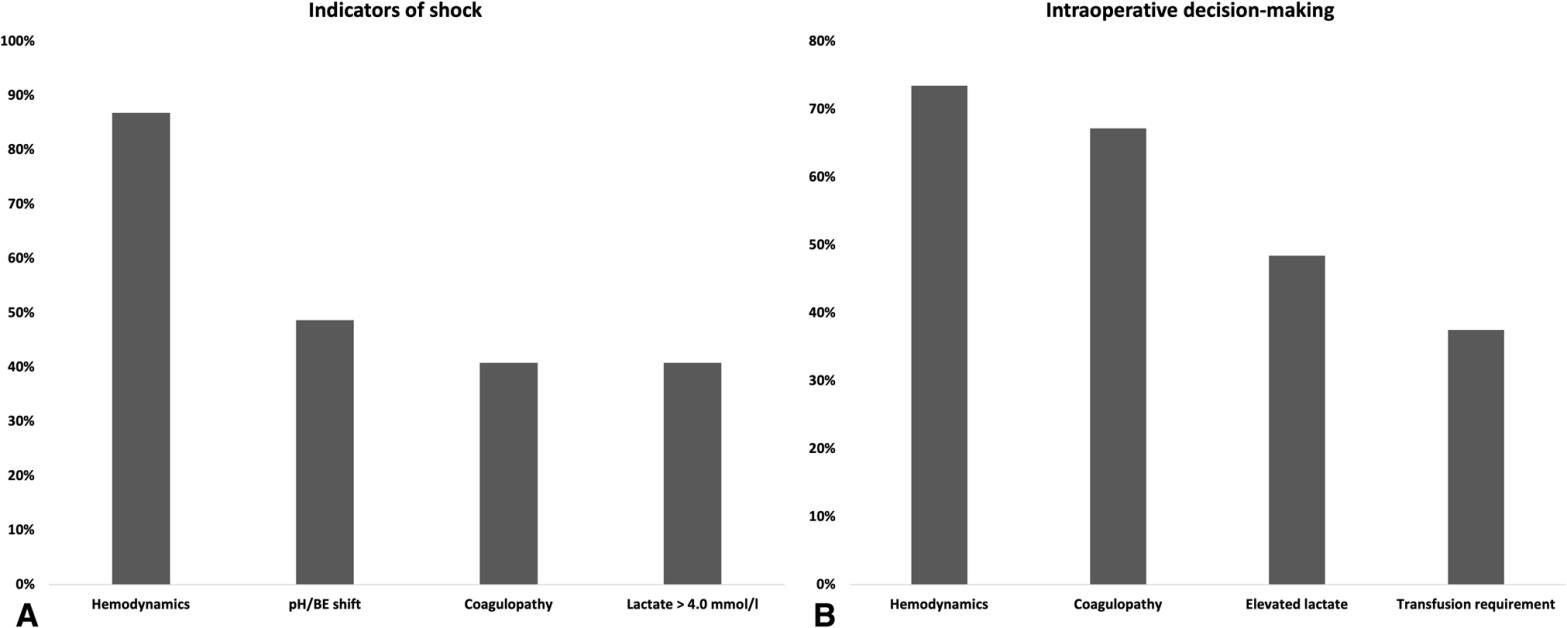
**A** Indicators of shock. **B** Intraoperative decision making during phase I

The 3 most relevant reasons to deem a polytrauma patient unstable (in shock) are depicted in Fig. 3a, b. Up to 86.8% (*n* = 66) mentioned a blood pressure < 90 mmHg on admission, the presence of coagulopathy (defined by ROTEM) (40.8%, *n* = 31) and acidosis (lactate > 4 mmol/l (40.8%, *n* = 31), and pH /BE shift (48.7%, *n* = 37). Similarly, regarding the intra-operative decision making, the majority of trauma surgeons rely on hemodynamic parameters (73.4%, *n* = 47), presence of coagulopathy (67.2%, *n* = 43), elevated lactate (48.4%, *n* = 31), and blood transfusion requirements (37.5%, *n* = 24).

### Secondary surgery

**Fig. 4:**
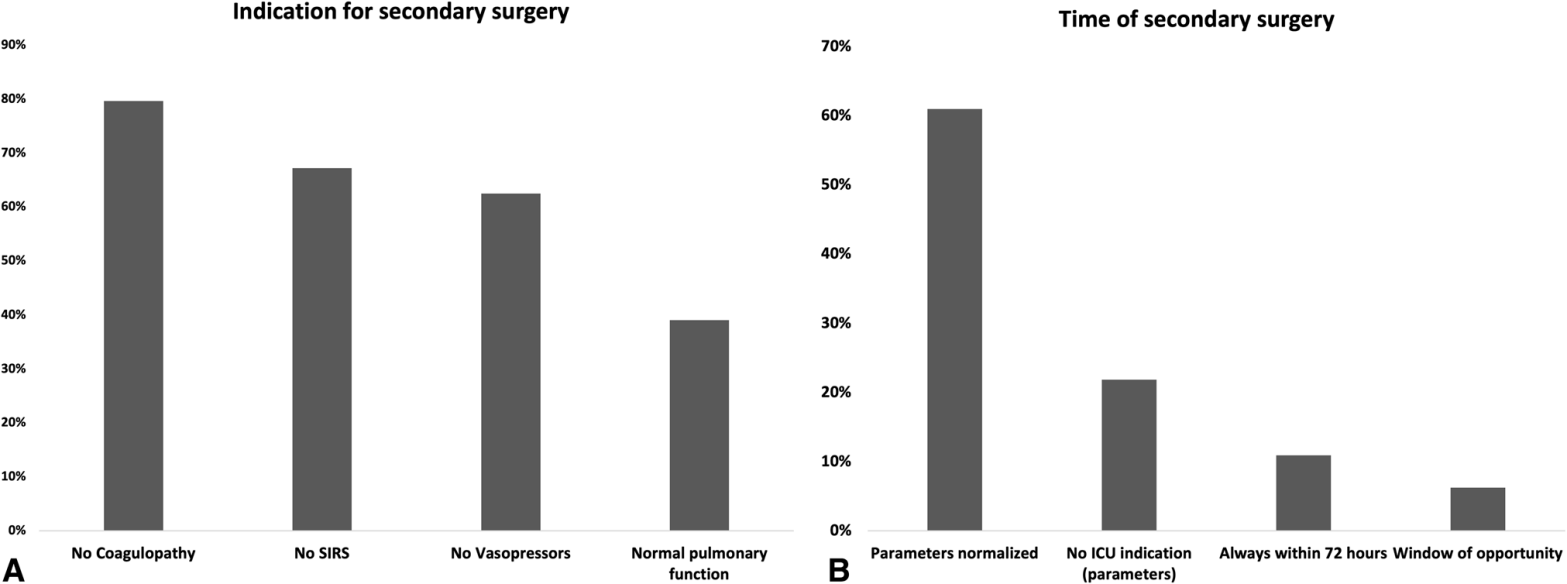
**A** Indication for secondary surgery. **B** Decision making to decide on the timing of secondary surgery

To clear the patient for secondary surgery after initial temporary stabilization, most of the surgeons check for absence of coagulopathy (76.7%, *n* = 51), absence of SIRS (67.2%, n = 43), and no need for vasopressors (62.5%, *n* = 40) (Fig. 4a, b). Regarding the timing of secondary surgery, 60.9% (*n* = 39) of the respondents operate after normalization of physiological parameters. Only the minority (6.3%, *n* = 4) of participants wait for the window of opportunity.

**Fig. 5:**
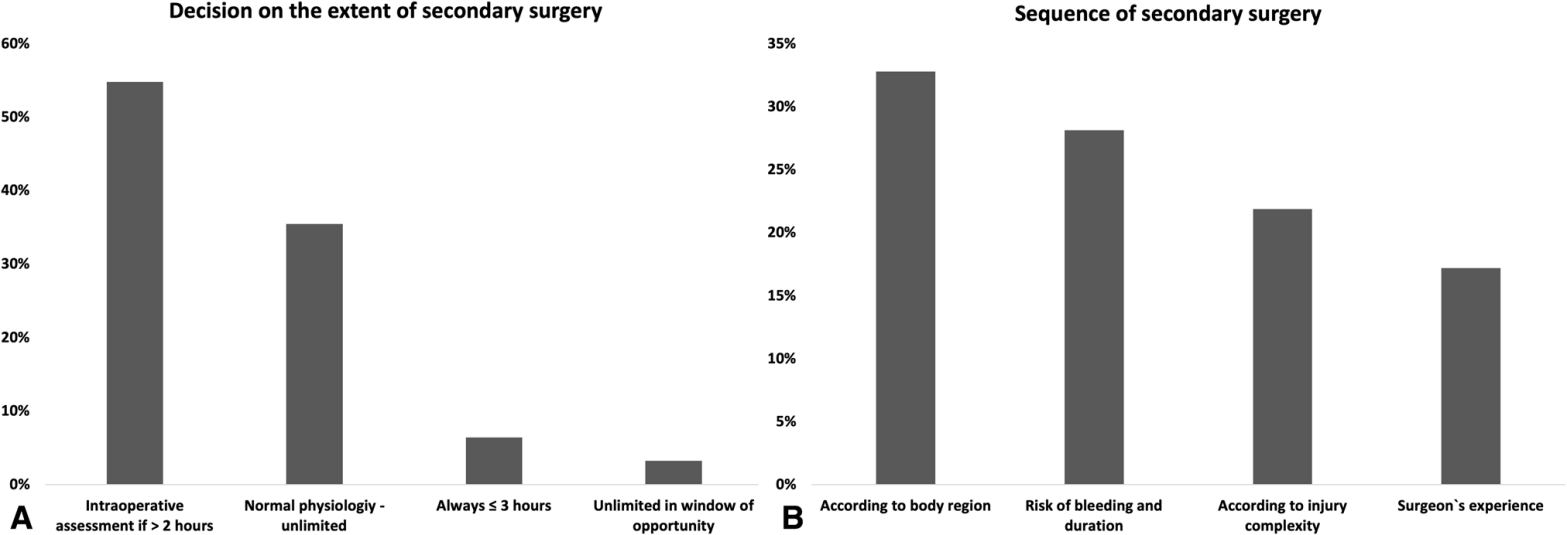
**A** Decision on the extend of secondary surgery. **B** Sequence of secondary surgery

In the presence of normal physiological parameters, the extent of the secondary surgery is unlimited (35.5%, *n* = 22). If the duration of the intervention is longer than 2 h, surgeons suggest an intraoperative evaluation of patients` hemostasis (54.8%, *N* = 34). There was no consensus in the trauma surgeon’s responses with regard to the sequence of the secondary surgery in stable multiply injured patients. Most surgeons (32.8%, *n* = 21) mainly decide the strategy according to injured body regions (e.g., trunk first, long bone second, etc.), while others (28.3%, *n* = 18) stratify according to the risk of bleeding and duration of the secondary intervention or according to the complexity (21.8%, *n* = 14) of the fracture (Fig. 5a, b).

## Discussion

Major trauma accounts for up to 20% of mortality after blunt injuries and is related to high morbidity rates [16, 17]. The stabilization of major fractures is a critical component of the care of these complex patients. [18]

The aim of the present study was to assess current fracture in polytraumatized patients among trauma surgeons in Europe.

In our survey, definitions of the polytrauma patient differed widely. The majority of participants used the anatomic injury severity score (ISS) for identifying polytrauma patients (ISS > 15) [19]. Twenty-five percent of the participants use the new “Berlin Definition,” and another quarter use definitions focusing on life-threatening conditions. Previous studies have indicated the lack of conformity in the definition of polytrauma [20, 21]. Our study findings agree with these previous studies since the European participants of the study used numerous polytrauma definitions. However, across Europe, it seems that three polytrauma definitions (ISS > 15, “Berlin definition” and “life-threatening”-definitions) are used predominantly. Several studies indicated that taking physiological parameters into account when defining polytrauma (such as the “Berlin definition”) can improve the identification of patients with higher risk of mortality and extended need of resources [22–24]. However, an abbreviated injury scale (AIS)-based anatomical definition is the most practical and feasible tool in daily practice. Of course, a uniform definition of polytrauma would allow reliable comparison of data and outcome parameters. Therefore, further consensus process might be needed to standardize the definition used in daily practice. Unfortunately, we could not differentiate in what kind of hospitals polytrauma treatment guidelines are unavailable. However, we feel that in hospitals with a high load, multiple injured patients’ protocols and guidelines should be present in order to improve the quality of treatment. Moreover, studies have shown that national guidelines on the treatment of polytrauma patients have helped standardize treatment procedures and have significantly improved the quality of care for severely injured patients [24, 25].

According to our results, most trauma surgeons defined physiologically unstable patients as those with compromised hemodynamics who are nonresponsive to resuscitation, with coagulopathy, and acidosis. This is in line with several previous studies, as multiple parameters are used [26–28]. Interestingly, in addition to physiological parameters, injury severity—as determined by the initial ISS score—seems to play a less relevant role in the present study. Furthermore, the mentioned abnormal parameters utilize the “lethal triad” (hypothermia, coagulopathy, and acidosis), and the majority of surveyed surgeons apply additional parameters down the line [14]. Regarding intra-operative decision making on the extent of surgery, trauma surgeons also relied on hemodynamics, coagulopathy, acidosis, and requirement for blood transfusion. To our knowledge, there are no available data on intra-operative decision-making strategies in polytrauma patients with regard to their physiological status. Still, it seems logical that these physiological parameters have some effect on the extent and duration of the procedure and therefore the patient’s outcome.

After temporary fixation and stabilization, planned secondary surgeries need to be timed. To clear polytrauma patients for secondary (definitive) surgery after initial temporary stabilization, most of the questioned trauma surgeons utilize normal hemodynamic parameters, absence of coagulopathy, and absence of SIRS (systemic inflammatory response syndrome) as indicators for definite fixation [12]. Accordingly, most of the participants set the timing of secondary surgery according to the normalization of the listed parameters. Contrary to these findings, a preset time plan based on a given day post-injury (usually after day 2–4; “window of opportunity”) has previously been considered the gold standard in the treatment of polytrauma [13]. Our study indicates that the physiological parameters are still of importance (absence of SIRS, coagulopathy). Still, the time frame of secondary surgery is not held so strictly anymore, and the window of opportunity loses relevance. When considering the extent of secondary surgery, more than one-third of the participants stated that there was no limit if physiological parameters remained within normal limits.

In our survey, the majority of trauma surgeons described that an intraoperative assessment is mandatory if secondary surgery lasts more than 2 h. In a previous study of more than 3000 polytrauma patients, secondary surgery that lasts more than 3 h was related to an increased probability of organ dysfunction [29]. Limitation to three hours was stated by less than 10% of the participants. It seems that decision making on the extent of surgery is again based on the physiological parameters and clinical stability rather than pre-defined times.

Most trauma surgeons stated that they would sequence their secondary surgeries according to the body region and less than 30% stratify according to the duration and risk of bleeding. This may be relevant, as these surgeries are usually not associated with severe bleeding, and their goal is usually to achieve optimal reconstruction of articular surfaces and/or alignment of major fractures. However, the authors feel that injuries contributing the most to bleeding and elevated inflammation markers should be addressed first to maintain physiological stability.

When discussing treatment strategies in musculoskeletal trauma, most authors have emphasized and prioritized the management of “major fractures” which historically focused on the long bones of the lower extremity [30]. In our survey, however, most surgeons considered any fracture that affects management as a “Major fracture” and refer to any injury in addition to long bones. Major fractures are not clearly defined in the literature, which may call for further consensus in the future. According to AIS (abbreviated injury scale), major fractures can be defined arbitrarily as AIS ≥ 3 (“serious injury”), which includes every fracture of the femur as well as at least open multi-fragment fractures of other long bones and stable open or unstable fractures of the pelvis [31]. A recent study on indications for MUST surgery (isolated musculoskeletal fractures) and DCO (polytrauma patients) found similar findings in defining fractures qualifying for temporary fixation and therefore can be considered major fractures [10]. This differentiation would clarify why a staged procedure was performed (local versus systemic conditions) and facilitate the interpretation of these studies.

The present study has certain limitations. It is well known that surveys have minor level of evidence, and the outcome of this study is directly connected to the participant’s understanding of the questionnaire. Furthermore, the response rate of almost 20% in this survey can be seen as a lack of representativeness possibly resulting in a nonresponse bias. In addition, we did not assess the participants’ current country of workplace, and therefore, we can only assume that most of the participants were from a European country (ESTES members). After initial pilot testing, no further validation of the questionnaire was performed.

## Conclusion

This study aimed to assess the current standards of fracture management among European trauma surgeons.

This study revealed four main results:

1. Different definitions of polytrauma and treatment guidelines are used in daily practice in Europe.
2. The triad of death (shock/acidosis, coagulopathy, and hypothermia) is used to indicate a damage control intervention.
3. Surgeons appear to consider any fracture that affects management as a “Major fracture,” e.g., relevant pelvic and spine injuries in addition to long bones.
4. Secondary surgeries are performed as soon as normal physiological parameters are present, and a set time frame (“window of opportunity”) is no longer required.

To our knowledge, no such findings have been described in the literature so far.

Despite the lack of a uniform European guideline for fracture management, we observed many similarities in treating these complex injuries among the respondents. The indication for staged surgical fixation (DCO) is made according to a stable hemodynamic status, absence of coagulopathy, lactate clearance, and most importantly, concomitant soft tissue injuries known to represent a risk (lung contusion, severe extremity soft tissue trauma, vascular injuries). The timing of secondary surgery also depends on the patient’s physiological parameters, assessed daily, while a fixed planning based on days after injury (“window of opportunity”) is no longer used.

### Supplementary Information

Below is the link to the electronic supplementary material.

Supplementary file1 (PDF 590 KB)

## Data Availability

Data are available upon reasonable request.
