# Supplementary material for: Standards of fracture care in polytrauma: results of a Europe-wide survey by the ESTES polytrauma section
Source: Eur J Trauma Emerg Surg. 2022 Oct 13;50(3):671–8. doi: 10.1007/s00068-022-02126-3 (PMC11249422; doi:10.1007/s00068-022-02126-3)
Supplement: Supplementary file 1 — Supplementary file1 (PDF 590 KB) [file 68_2022_2126_MOESM1_ESM.pdf]

European Society for Trauma and Emergency Surgery — ESTES

USZ Universitäts Spital Zürich

Standards in Polytrauma Treatment: A Europe-wide survey

Demographics

1. What is your gender?

- ○ female
- ○ male
- ○ unspecified

2. What is your current level of education/training?

- ○ Intern
- ○ Resident
- ○ Attending
- ○ Senior Attending
- ○ Head of Department

3. How many years of work experience do you have?

0 ——————————————— 40 ☐

4. How many polytraumatized patients do you treat per month?

0 ——————————————— 100 ☐

Standards in Polytrauma Treatment: A Europe-wide survey

Diagnostics

6. What kind of poly trauma definition is used in your clinic?

1 Answer

- ◯ Polytrauma "Berlin Definition"

  (2 injuries greater or equal AIS 3 and one or more additional diagnosis (pathological condition): hypotension (syst. BP £ 90 mmHg,) unconsciousness (GCS £ 8), acidosis (base deficit £ -6.0), coagulopathy (PTT ≥ 40 sec. or INR ³ 1.4), age (≥ 70 years).)
- ◯ ISS ≥ 16
- ◯ ISS ≥ 25
- ◯ At least 2 severe injuries of the head, chest or abdomen or one of them in association with an extremity injury
- ◯ Another regional/internation definition? (please explain):

7. What are the **3** most relevant indicators for an unstable patient / patient in shock?

3 Answers

- ☐ Admission blood pressure < 90 mmHg (sys) / tachycardia / vasopressors
- ☐ Pathological Shock Index
- ☐ Coagulopathy (platelets < 90.000)
- ☐ Coagulopathy (by ROTEM)
- ☐ Lactate > 2.5 mmol/l
- ☐ Lactate > 4.0 mmol/l
- ☐ pH/BE shift

8. Which polytrauma treatment guidelines are used in your clinic?

1 Answer

- ◯ S-3-Guideline (by DGU®)
- ◯ Local hospital guideline
- ◯ Regional guideline
- ◯ Other international guideline
- ◯ No guideline

9. What is the role of thromboelastography (ROTEM® or TEG ®) in decision-making for Damage Control Surgery in your clinical practice?

1 Answer

- ◯ Very important
- ◯ Not relevant
- ◯ We do not use thromboelastography / we use other parameters

Indication

10. How would you define „major fractures" in polytrauma?

1 Answer

- ◯ According to body region: Femur, Pelvis, Spine, etc.
- ◯ Presence of a complex fracture situation (e.g. articular fractures AO-Type C)
- ◯ Presence of concomitant severe soft tissue injuries
- ◯ Degree of contamination of bone and soft tissues
- ◯ Presence of concomitant injuries (e.g. vascular or neurological injuries)
- ◯ Combination of the above answers

11. What is the most important indication for «MusculoSkeletalTemporary (MuST) Surgery *»?

1 Answer

*(MusculoSkeletal Temporary (MuST) Surgery = temporary stabilization of musculoskeletalinjuries in hemodynamicaly and physiologicaly stable patients)

- ◯ Presence of complex articular fractures
- ◯ Severe soft tissue injuries
- ◯ Severe wound contamination
- ◯ Surgeon`s experience
- ◯ Infrastructure of the hospital and / or the trauma system

Intervention

12. When do you use REBOA* in your setting ?

Several answers possible

* (resuscitative endovascular balloon occlusion of the aorta)

- ☐ To control all non-compressible major bleeding
- ☐ To control bleeding in blunt hemodynamically unstable trauma patients with pelvic fractures
- ☐ In truncal injuries proximal to pelvis
- ☐ We do not use REBOA

13. What is the most commonly used „damage control" intervention in lower extremity injuries?

1 Answer

- ○ External fixation
- ○ Traction
- ○ Both, external fixation and traction
- ○ Cast / Splinting

14. What is the standard sequence of femoral fracture fixation in your clinic in „unstable" patients?

1 Answer

- ○ 1st. External Fixation, 2nd unreamed intramedullary nailing
- ○ 1st. External Fixation, 2nd reamed intramedullary nailing
- ○ 1st. External Fixation, 2nd ORIF by plating
- ○ Definitive osteosynthesis with plating
- ○ Definitive osteosynthesis with nailing

15. Which parameters (max. 3) are most relevant for intra-operative decision-making in major surgery?

3 answers possible

- ☐ Coagulopathy (clinical signs / ROTEM)
- ☐ Urinary output
- ☐ Lactate levels / BE
- ☐ Hemodynamic stability / need for vasopressors
- ☐ Drop in body temperature
- ☐ Transfusion / blood loss
- ☐ Duration of surgery > 2 h
- ☐ Duration of surgery > 6 h

Secondary surgery

16. What are the most relevant (max. 3) parameters used to clear the patient for secondary surgery after a polytrauma?

3 answers possible

- ☐ Absence of SIRS criteria
- ☐ Normal / negative input/output ratio
- ☐ Normal pulmonary function
- ☐ No relevant administration of vasopressors
- ☐ Absence of coagulopathyt

17. When do you perform secondary surgery after polytrauma?

1 Answer

- ○ As soon as the parameters have normalized
- ○ If ICU clears by parameters
- ○ Always within 72 hours
- ○ Always wait for the window of opportunity (5 days)

18. How do you decide on the extend of the secondary surgery?

1 Answer

- ◯ Always limit to 2-3 hrs.
- ◯ If parameters are normal, unlimited duration
- ◯ Assess intraoperatively, if surgery > 2 hours
- ◯ No limit after window of opportunity (wait 5 days)

19. How do you group the sequence of secondary surgery in stable multiple injured patients?

1 Answer

- ◯ According to body region (trunk first, long bones second, etc.)
- ◯ According to the complexity of fractures
- ◯ Risk of bleeding and duration of operation
- ◯ Experience of the surgeon or status of infrastructure
